# Early Rotem Evaluation Abnormalities May Be Associated with Hemorragic but Not Thromboembolic Complications in Patients with Major Trauma Referred to Hospital Within the First Hour

**DOI:** 10.3390/jcm15166315

**Published:** 2026-08-15

**Authors:** Carlo Rostagno, Celeste Marchetti, Andrea Nencioni, Alessandro Coppa, Federica Guerra, Lara Gianesello, Simone Vanni

**Affiliations:** 1 Dipartimento Medicina Sperimentale e Clinica, Università di Firenze, 50121 Firenze, Italy; cele.marchetti97@gmail.com (C.M.); simone.vanni@unifi.it (S.V.); 2 Medicina e Chirurgia d’Urgenza e Accettazione, AOU Careggi, 50134 Firenze, Italy; nencionian@aou-careggi.toscana.it (A.N.); coppaa@aou-careggi.toscana.it (A.C.); guerraf@aou-careggi.toscana.it (F.G.); 3 Anestesia e Rianimazione per Ortopedia, AOU Careggi, 50134 Firenze, Italy; gianesellol@aou.careggi.toscana.it

**Keywords:** trauma, hemostasis, bleeding, venous thromboembolism

## Abstract

**Background**: Trauma-induced coagulopathy is associated with a high rate of hemorrhagic complications and with increased mortality after major trauma. Viscoelastic techniques have been demonstrated to be useful for early diagnosis and appropriate management in these patients. **Methods**: This prospective observational study included patients who underwent major trauma (ISS (injury severity score) > 15) and were referred to hospital within the first hour. All underwent early ROTEM evaluation. **Results**: The study included 100 patients (80 males, 20 females, mean age 52.41 years). Twenty-three had bleeding, requiring transfusion (mean age of 57.3 years and ISS 21.7). According to accepted criteria of hypocoagulability, 10/23 (43.5%) had at least one abnormality in one of the ROTEM parameters. EXTEM CT was significantly longer in transfused patients. Four had symptomatic VTE (venous thromboembolism), and Doppler examination showed DVT (deep venous thrombosis) in 14 (14%) of the population. Sepsis, pneumonia, and acute renal failure were statistically more frequent in patients with thrombosis, while no relation was found with ROTEM. Finally, 3 out of 5 patients who died showed a severe hypocoagulable condition at initial ROTEM evaluation. **Conclusions**: In patients with major trauma referred to hospital within the first hour, early qualitative ROTEM examination at admission may be useful in identifying patients at bleeding risk. Sepsis, pneumonia, and acute renal failure, but not ROTEM parameters, were associated with the risk of thromboembolic events.

## 1. Introduction

Head trauma, hemorrhage and trauma coagulopathy are the more frequent causes of death in trauma patients [1]. Severe trauma, regardless of the location, may trigger a systemic complex response characterized by the release of pro-inflammatory cytokines, complement activation, and neutrophil recruitment [1,2]. Moreover, a damaged endothelium releases pro-hemorrhagic and pro-thrombotic factors [3]. This systemic reaction may be responsible for trauma-induced coagulopathy (TIC). This is characterized by clotting derangement, with an early hypocoagulable state favoring excessive bleeding and/or a later hypercoagulable state that may lead to thromboembolic events and multiple organ failure [4,5,6]. Trauma coagulopathy is the leading cause of death from bleeding after fatal hemorrhage due to vascular injury, with a mortality rate of 30–50% [2,7,8]. Moreover, patients who develop TIC receive a higher number of transfusions, spend more time in intensive care, undergo more days on mechanical ventilation, and have a higher incidence of multi-organ dysfunction [9]. Viscoelastic techniques are an essential tool for early diagnosis and appropriate management of trauma coagulopathy [6,10,11]. The prospective RETIC study showed the advantages of using point-of-care thromboelastometry in the choice of a specific correction therapy [12]. In the iTACTIC multicenter trial, 24 h mortality was lower in patients treated with a Massive Transfusion Protocol optimized by viscoelastic techniques [13]. In addition, it has been demonstrated that TEG/ROTEM-guided transfusions are associated with a decreased need for endoscopic or surgical embolization, and with a reduced risk of acute renal failure [14]. Nevertheless, all these studies examined non-homogeneous populations, with different ISS, timing of viscoelastic examination and highly variable prehospital fluid treatment with potential bias in result interpretation. The aim of the present study was to evaluate the relation between thromboelastic parameters and the development of thrombotic and hemorrhagic complications in severe trauma patients (ISS >15) referred to hospital within the first hour from trauma, thus limiting the effects of hemostatic changes related to fluid treatment and time. The endpoints were the relation between ROTEM abnormalities at admission, the need for transfusion, as a surrogate of hemorrhagic complications, and the development of deep vein thrombosis and pulmonary thromboembolism. The secondary endpoint was in-hospital mortality.

## 2. Materials and Methods

The study is part of the Finalized Research Project 2018 (NET-2018-12368077) of the Ministry of Health. It was approved by the Regional Ethics Committee for Clinical Trials of the Tuscany Region, section Area Vasta Centro (n 13556_oss), on 2 June 2020. Informed consent was obtained at admission by patients or by their legal tutors. The study was conducted according to STROBE statements and performed in line with the principles of the Declaration of Helsinki.

### 2.1. Population of Study

The prospective observational study included patients aged >18 years, referred to the Emergency Department of AOU Careggi from 1 January 2023 to 31 December 2023, within the first hour after major trauma (ISS > 15). Patients in therapy with antiplatelet or anticoagulant drugs (direct oral anticoagulants, anti-vitamin K) were excluded from the study. A few patients were not included due to a lack of personal data or direct discharge by the Emergency Department. Standard prophylaxis of VTE with enoxaparin 4000 UI was started at admission in patients without significant brain injury (n = 48), within 48 h in the other 37, while pneumatic compression was used in the last 15 patients until anticoagulation was considered safe. None underwent caval filter insertion.

### 2.2. Study Design and Data Collection

The aim of the study was to evaluate the relation between ROTEM abnormalities at admission and the need for transfusion. We used transfusion as a surrogate for bleeding since it was the only objective measurable parameter. Other factors, including surgery, may influence the need for transfusion. However, most emergency surgery in these patients was performed for bleeding control and was often associated with the severity of trauma. Moreover, we evaluated the relation between ROTEM parameters and the development of asymptomatic deep vein thrombosis and of symptomatic pulmonary thromboembolism. Finally, the secondary endpoint was in-hospital mortality.

The transfusion threshold was set at Hb values below 7 g/dL and/or hemodynamic instability. Compression ultrasonography, using the standard B-mode method (CUS), was performed on all patients on the 5th postoperative day and every three days thereafter. Portable Doppler ultrasonography (Alpha, Esaote, Firenze, Italy) with high-resolution linear probes (5–7.5 MHz) was used for examination.

The severity of trauma was assessed according to the Injury Severity Score (ISS). In the Emergency Department, blood was collected for blood count, coagulation (PT, aPTT, and fibrinogen), creatinine, D-dimer, Reactive C Protein, CK, and gas analysis. Moreover, External (EXTEM) and Internal (INTEM) coagulation pathways and Fibrinogen function (FIBTEM) were explored by Coagulation Time (CT, in seconds), Clot Formation Time (CFT, in seconds), A5 (MCF amplitude after 5 min from the end of the CT, in mm), A10 (MCF amplitude after 10 min), and Maximum Clot Firmness (MCF, in mm) using ROTEM System (Werfen, Monza, Italy). In agreement with the accepted criteria, a condition of hypocoagulability was associated with the following parameters: EXTEM CT > 80 s, an EXTEM A10 < 40 mm, an EXTEM MCF < 30 mm, and a FIBTEM A10 < 7 mm [15]. Hypercoagulability parameters were considered an EXTEM CT < 40 s and an EXTEM A10 > 60 mm [16]. Infectious complications, including sepsis, acute renal failure, and cardiopulmonary complications other than episodes of delirium, were recorded.

### 2.3. Statistical Analysis

The statistical analysis was performed using the SPSS (Statistical Package for Social Sciences, Chicago, IL, USA) software, version 25.0. Continuous parameters were reported as mean values and standard deviation. Differences between groups were evaluated using Student’s *t* test. For non-parametric variables, the Wilcoxon test was used. For categorical parameters, any difference between groups was evaluated using the Pearson chi^2^ test or the Fisher test when indicated. Linear regression analysis was used to relate standard hemostatic tests and ROTEM variables. Multivariate logistic regression analysis was performed to identify independent prognostic factors.

## 3. Results

**Figure 1:**
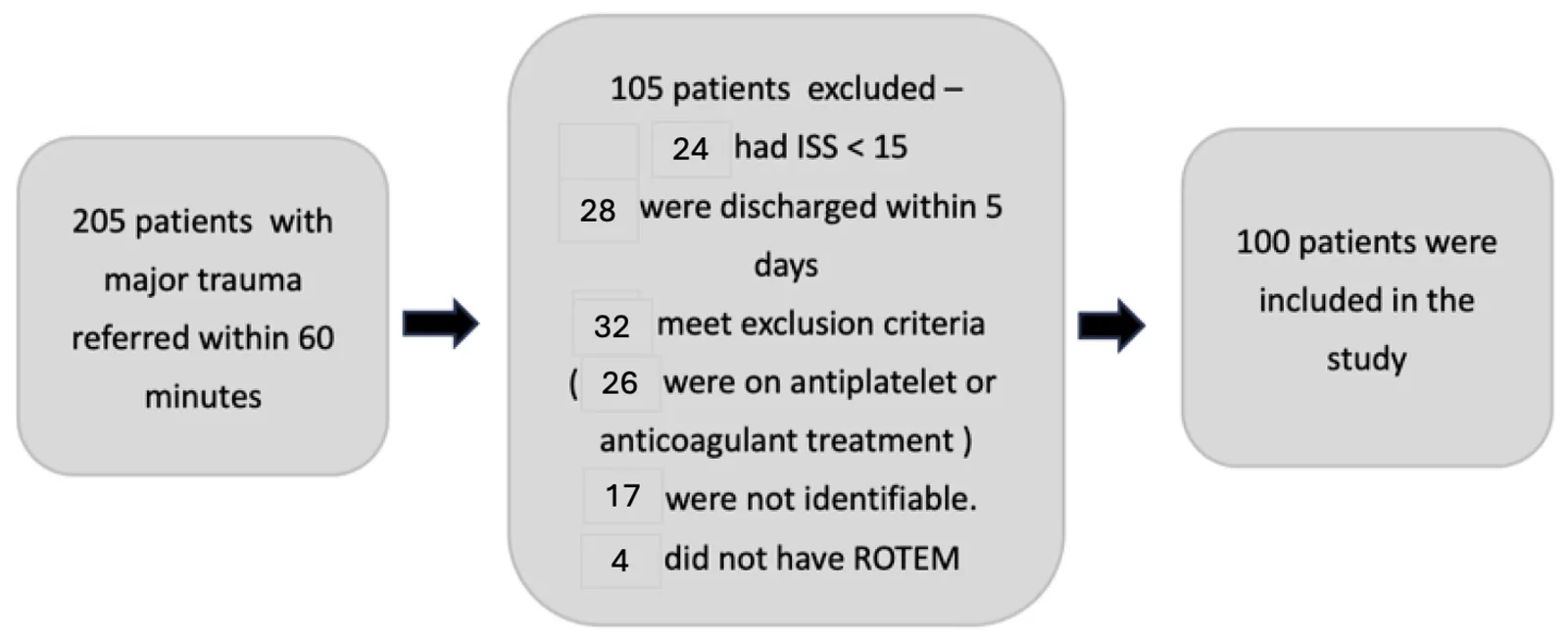
Flow chart of the study.

The study included 100 (80 males, 20 females, mean age 52.41 years) out of 205 patients with polytrauma or major trauma admitted to the Emergency Department of the A.O.U. Careggi from January 1 to 31 December 2023. The mean Injury Severity Score was 21 points. A major comorbidity before trauma (previous coronary revascularization) was found in one patient. Figure 1 reports the flow chart for admission to the study.

Only one patient received a crystalloid infusion of >1500 mL before hospital admission, eight (8%) received between 1000 and 1500 mL, and only one patient required vasoactive amines. Control of pain and sedation was needed in almost all patients. Following blood collection for ROTEM analysis, all patients received tranexamic acid. In Table 1 are reported blood count, hemostatic parameters, and ROTEM results at admission. Seventy-two patients underwent surgery, six in emergency, 31 in urgency, and the other as deferred procedures in relation to the type of trauma and/or general clinical conditions. In 55 cases, surgery was performed under general anesthesia. The average time between admission to hospital and surgery was 21 +/− 73 h. The mean length of hospital stay was 23.3 +/− 20.2 days. Four of the patients enrolled in the study had symptomatic VTE. Doppler examination on the fifth day of hospitalization showed DVT in 14% of the population. Twenty-three patients required one or more packed RB cell transfusions. Sepsis complicated clinical course in 26% of the population, respectively. Five patients died during their hospital stay.

### 3.1. Relation Between ROTEM Parameters and Coagulation Parameters

**Figure 2:**
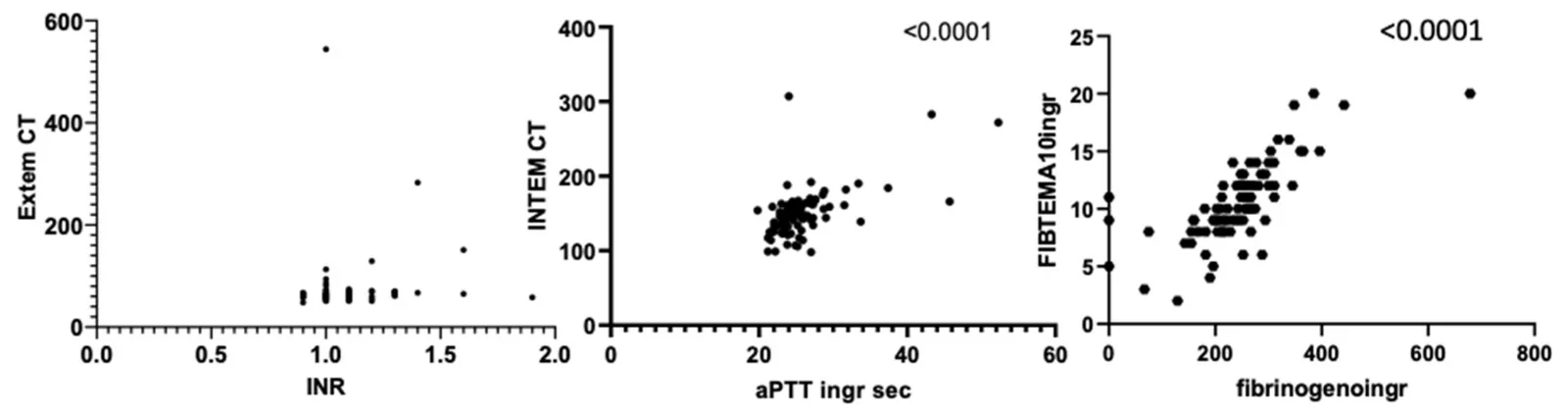
Relation between ROTEM parameters and coagulation parameters.

An INR of above 1.2 was detected in 10 patients. Of these, three had less than 120,000 platelets, and four had fibrinogen values below 150 mg/dL. We did not find any relation between INR and EXTEM CT values; aPTT values were significantly related to INTEM CT values. Finally, a statistically significant correlation existed between fibrinogen concentrations and FIBTEM A10 values (Figure 2).

### 3.2. Abnormalities of ROTEM Parameters

An EXTEM CT value > 80 s was detected in 8/100 patients, an EXTEM A10 < 40 mm in 3/100 patients, an EXTEM MCF < 30 mm in none, and finally, a FIBTEM A10 < 7 mm was found in 10/100 patients. Ten out of 23 patients transfused (43.5%) had at least one abnormality in one of the ROTEM parameters.

Regarding hypercoagulability cut-offs, no patient showed an EXTEM CT < 40 s, while an EXTEM A10 > 60 mm was detected in 12 patients.

### 3.3. Hemorrhagic Complication

Twenty-three (23%) patients had bleeding requiring transfusion (mean age of 57.3 years and mean ISS 21.7). Twenty-one (21%) were transfused in the first 24 h from trauma. Seventeen (17%) patients needed at least two RB cell units. In this group four patients needed fresh frozen plasma and three platelet apheresis. Four patients were transfused due to hemodynamic instability, and the other 19 for Hb values below 7 g/dL. We did not find significant difference in nadir Hemoglobin between transfused and non-transfused patients. Patients with bleeding complications more frequently underwent emergency/urgency surgery (65% vs. 31%, *p* = 0.0005). Time to surgery was 0.9 ± 1.9 days in patients needing transfusion vs. 2.2 ± 3.6 days (*p* = 0.007) in patients who did not. EXTEM CT (Clotting Time of the extrinsic coagulation pathway) was significantly longer in transfused patients (*p* < 0.0001) (Table 2).

None of the other ROTEM or hemostatic parameters showed a statistically significant difference between the two groups. Moreover, there was no difference in the main clinical complications.

**Figure 3:**
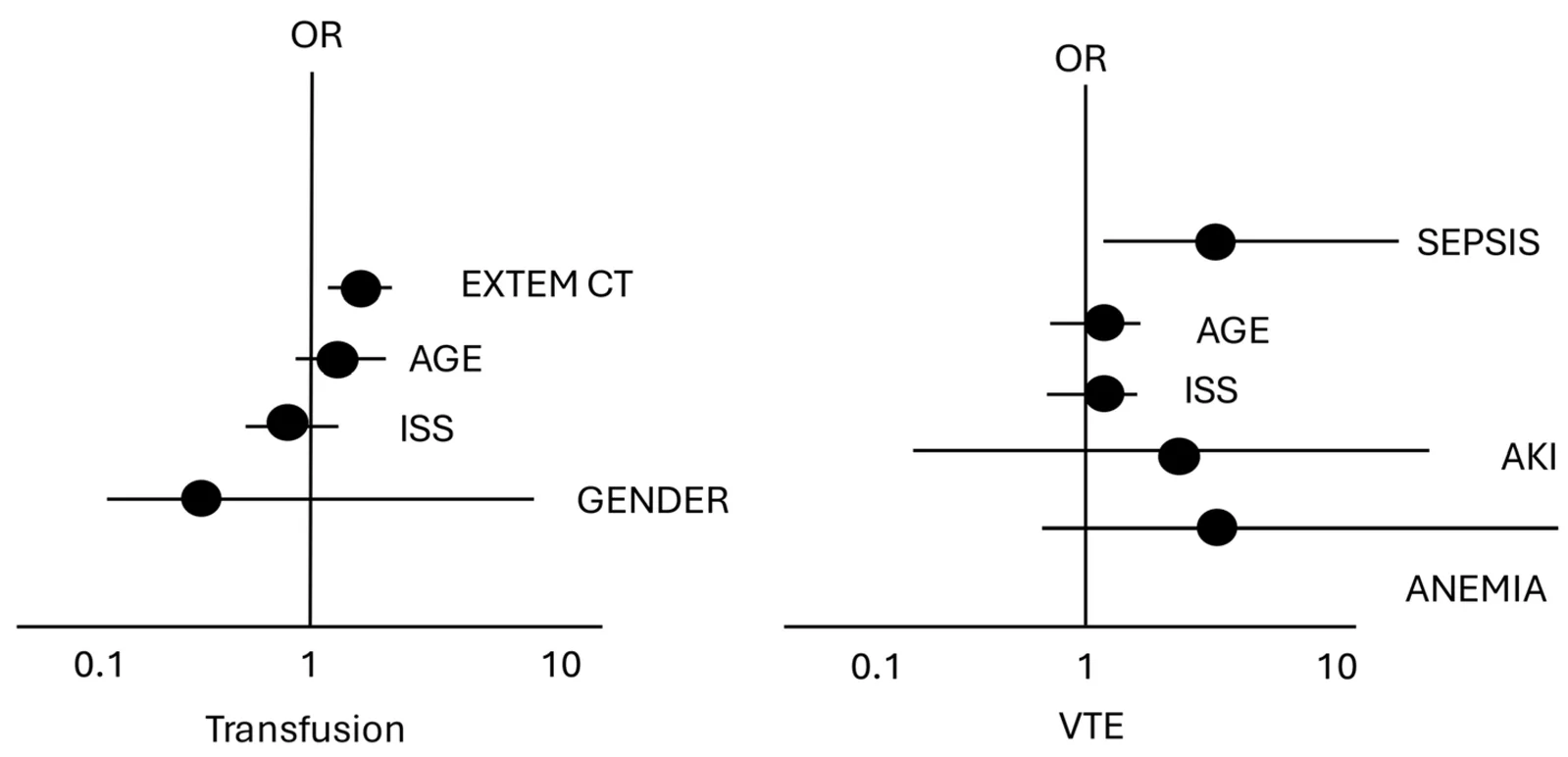
Multivariate analysis of need for transfusion and venous thromboembolism. (ISS, injury severity scor AKI, acute kidney injury).

At logistic multivariate analysis only EXTEM CT (OR 1.14, 95% CI 1.0–1.20, *p* = 0.04) (Figure 3) was associated with the need for transfusions.

### 3.4. Thromboembolic Events

Fourteen patients developed thrombotic complications. Ten had Doppler documented proximal DVT within the 5th day of hospitalization. Four had symptomatic VTE, three within the 5th day, one on seventh day after admission. No relationship was found between VTE and the timing and type of prophylaxis, nor was enoxaparin associated with hemorrhagic events. We did not find any significant difference in the ROTEM parameters between the two groups (Table 3).

### 3.5. Hospital Mortality

Five patients died during hospitalization. They were older (mean age 67 years vs. 51.64 years, *p* = 0.005) and with a more severe trauma (ISS 49.2 ± 15 vs. 19.2 ± 4.1, *p* < 0.0001) in comparison to patients discharged alive. In Table 4 are summarized some characteristics, blood counts, and ROTEM values of deceased patients. 3/5 (60%) patients who died had at least a parameter of hypocoagulability at ROTEM. Three out of ninety-five patients (3.2%) discharged alive had an EXTEM CT > 80 s in comparison to 3/5 patients (60%) who passed away (*p* 0.002). Similarly, 2/5 (40%) deceased patients had an INTEMCT > 240 s, compared to only 1/95 (1,1%) of the survivors (*p* = 0.006). Finally, 3/5 (60%) deceased patients had a FIBTEM A10 < 7 mm, in comparison to 7/95 (7.3%) patients discharged alive (*p* = 0.007).

## 4. Discussion

Trauma coagulopathy (TIC) is a threatening complication of major trauma [16]. In the last decade, the pathogenetic mechanisms have been defined [17]. Tissue hypoperfusion triggers an exaggerated activation of protein C mediated by the Thrombomodulin pathway, leading to severe hypocoagulability [18]. Bleeding risk is also associated with increased fibrinolytic activity, in part related to the inhibition of Plasminogen Activator Inhibitor-1 (PAI-1) by protein C [19]. In the post-resuscitation phase, there is an excessive response that leads to a prothrombotic state, predisposing to thromboembolism. There is no commonly accepted definition of TIC based on thromboelastometric data. An increase in clotting time and clot formation time (CT and CFT), loss of clot width (CA), and maximum clot width are suggestive of an alteration in hemostasis. The incidence of trauma-induced coagulopathy is related to the selected population, the time between trauma and hospitalization, the quantity and type of fluids administered before hospitalization, and several other variables. Early-stage coagulopathy has been described in 15–30% of trauma patients and is related to the severity of trauma expressed as the Injury Severity Score. The mortality associated with trauma coagulopathy ranges from 5 to 40% [4,20,21]. Several studies suggested the usefulness of viscoelastic techniques for early diagnosis and appropriate management of trauma coagulopathy [6,10,11] with a lower early mortality in patients treated with a Massive Transfusion Protocol optimized by ROTEM results (14), a decreased need for endoscopic or surgical embolization, and a decreased incidence of acute renal failure. A recent metanalysis, however, questioned the sensitivity of thromboelastometry in the diagnosis of trauma coagulopathy [22]. Moreover, the predictive utility of these tests may be limited to the need for blood-product transfusion and not related to mortality and other outcomes [23]. The patients included in our study all had severe trauma; the average ISS value was 21, and 55/100 had trauma involving the skull, skeletal segments, and internal organs. Hospitalization times were on average less than 1 h from the time of the trauma, and only one patient underwent crystalloid infusion >1500 mL. The need for transfusion was considered a surrogate endpoint for bleeding complications. According to the guidelines [15], patients were transfused for Hemoglobin values below 7 g/dL, except for patients who were in hypovolemic shock despite higher Hb values. Of the 23 patients who required transfusion therapy, 10 (43.5%) had one or more ROTEM alterations, suggesting hypocoagulability. Moreover, EXTEM CT was significantly longer in patients requiring transfusions. Standard hemostatic parameters did not have any predictive value, with a large overlap between groups. Hagemo et al. [24] described 808 patients with trauma (mean ISS 30) with characteristics like those included in our study (hospital arrival time < 2 h and crystalloid infusion less than 2000 mL). Transfusion of four or more RBC units was required in 6.1%, while 11.0% had ROTEM abnormalities suggestive of TIC. These values are not significantly different from those found in our study, in which 7% needed four or more RBC units, while 10% had signs of hypocoagulability. The number of RBC and plasma units transfused was higher for EXTEM A5 values below 35 mm and FIBTEM A5 < 8 mm. An Australian study showed that pre-hospital coagulation dysfunction was associated with mortality and early massive transfusion [25]. In our study three out of five patients who died in hospital had clear signs of trauma coagulopathy with severe ROTEM abnormalities at admission. They were on average older (mean age 67.5 years) and with a high mean ISS score (49.3) in comparison to patients discharged alive. The limited number of events does not allow us to draw strong conclusions that should be supported by larger studies. Thromboembolic complications were more frequent in patients with infective complications and acute renal failure. None of the ROTEM parameters showed significant differences in patients with or without thromboembolic complications. At the logistic multivariate analysis, EXTEM CT was associated with transfusion while the development of sepsis predicted venous thromboembolic complications. Nevertheless, the lack of relation between early ROTEM parameters and DVT may reflect the temporal evolution of trauma-induced coagulopathy since the shift toward a hypercoagulable state typically occurs several days after injury.

## 5. Limitations

It should be stressed that the study has several limitations. The first is represented by the size of the sample and the consequent relatively low incidence of hemorrhagic and thrombotic complications. Due to a delay in patient enrollment related to the COVID pandemic, we chose to reach 100 eligible patients to respect the times imposed by the ministerial project, without formal sample size with statistically power calculation. Overall, the number of clinical events and the mortality (5%) were relatively low considering the average severity of the trauma (mean ISS 21). Therefore, statistical analysis may be mainly explorative. We used transfusion, with a threshold of Hb < 7 g/dL and/or hemodynamic instability, as a surrogate for hemorrhagic complications, and this may in part reflect injury severity and the need for emergency surgery rather than coagulation status. Nadir Hemoglobin was not a reliable endpoint. We did not find significant differences between transfused and non-transfused groups. Moreover, the nadir was reached at different times after trauma and was influenced by late surgical interventions aimed to correct anatomic damage more than to limit early bleeding, other than infective complications and degree of hemodilution.

Another criticism may be that the single-point ROTEM examination may have overlooked dynamic changes. It must be emphasized that previous investigations did not show significant changes in serial ROTEM samples performed after trauma [24]. In our opinion, the early ROTEM evaluation at hospital admission, and on average less than an hour after the trauma, may be a point of strength of the study, allowing us to limit the confounding effects related to the amount of fluid administered or to prolonged hypothermia. ROTEM changes may more clearly reflect trauma-induced clotting abnormalities.

## 6. Conclusions

Data of the present study suggest that, in patients with major trauma, the ROTEM examination at hospital admission may be useful to intercept patients requiring transfusion. Signs of hypocoagulability in fact are found in about 40% of transfused patients. Several factors, in particular the occurrence of sepsis, pneumonia, and acute renal failure, all associated with an increased length of hospitalization, seem to correlate with the risk of thromboembolic events.

## Figures and Tables

**Table 1:** Main laboratory examination and average ROTEM parameters at admission.

Hemoglobin g/dL	13.6 ± 1.9	INTEM CT	149 ± 32	EXTEM CT	71 ± 54	FIBTEM CT	91 ± 47
Hematocrit (%)	39.5 ± 5.6	INTEM CFT	77± 30	EXTEM CFT	81 ± 63	FIBTEM CFT	34 ± 54
Platelets 10^3^ μL	231 ± −67	INTEM A5	44 ± 6	EXTEM A5	44 ± 7	FIBTEM A5	9 ± 3
A PTT sec	25.8 ± 4.8	INTEM A10	53 ± 6	EXTEM A10	54 ± 7	FIBTEM A10	10 ± 3
Fibrinogen g/dL	244 ± −89	INTEM MCF	60 ± 5	EXTEM MCF	63 ± 5	FIBTEM MCF	11 ± 3

(CT: clotting time, CFT: clotting formation time, A5: amplitude at 5 min, A10: amplitude 10 min, MCF: maximum clot firmness).

**Table 2:** Comparison of transfused and not-transfused patients.

	Transfused n = 23	Not Transfused n = 77	*p*
Age (years)	57.3 ± 12	51.1 ± 17	0.10 *
ISS (injury severity score)	21.7 ± 5.6	20.5 ± 5.1	0.31 *
Hemoglobin g/dL	12.1 ± 2.3	13.9 ± 1.6	0.001 *
Hemoglobin nadir g/dL	7.2 ± 2.6	7.9 ± 4.1	0.15
Hematocrit %	36.3 ± 7.2	40.4 ± 4.6	0.01 *
Platelets 10^3^ μL	227 ± 70	233 ± 70	0.05 *
aPTT sec	27 ± 7	25 ± 4	0.1 *
Fibrinogen mg/dL	244 ± 51	248 ± 74	0.8 *
Extem CT sec	97 ± 12	63 ± 12	0.0001 *
Extem A10 mm	53.1 ± 10	55 ± 5	0.2 *
Intem CT sec	160 ± 46	145 ± 27	0.07 *
Intem A10 mm	53 ± 7	54 ± 6	0.5 *
Fibtem A10 mm	10 ± 4	11 ± 3	0.2 *
Pneumonia	8	16	0.17 #
Respiratory failure	13	31	0.16 #
Wound infection	5	9	0.22 #
Acute renal failure	5	5	0.04 #
Delirium	4	6	0.2 #
Sepsis	8	18	0.27 #

* Student’s *t* test, # chi square test. (CT: clotting time, A10: amplitude 10 min).

**Table 3:** Comparison of patients with and without thromboembolism.

	DVT n = 14	No DVT n = 86	*p*
Age (years)	57.2 ± 16.8	51.5 ± 17.4	ns
ISS (injury severity score)	22.4 ± 5.4	20.5 ± 6.1	ns
Hemoglobin g/dL	14.1 ± −2	13.5 ± 1.9	ns
Hematocrit %	41.1 ± 5.4	39.4 ± 5.6	ns
Platelets 10^3^ μL	207 ± 50	236 ± 69	ns
aPTT sec	25.52 ± 5.51	25.9 ± 4.8	ns
Fibrinogen mg/dL	237 ± 53	245 ± 94	ns
Extem CT sec	84 ± 59	68 ± 53	ns
Extem A10 mm	52.79 ± 0.46	54.8 ± 6.8	ns
Intem CT sec	153.6 ± 39.9	148.1 ± 31.5	ns
Intem A10 mm	51.21 ± 6.5	54.2 ± 6	ns
Fibtem A10 mm	9.6 ± 3.5	10.6 ± 3.3	ns
Pneumonia	7	17	0.037
Wound infection	4	10	ns
Acute kidney failure	4	6	0.032
Sepsis	8	18	0.008
Transfusion	5	8	ns

ns: not statistically significant.

**Table 4:** Characteristics of patients who died in hospital. (LOS = length of stay).

Age Years	Gender	ISS	LOS (Days)	INR	Fibrinogen g/dL	EXTEM CT	EXTEMA10	FIBTEM A10
86	Male	46	6	1.4	129	283	36	2
58	Male	41	12	0.9	442	66	64	19
77	Male	75	3	1.6	67	151	36	2
50	Female	48	2	1.3	159	67	54	9
64	Male	34	3	1	182	94	43	6

## Data Availability

Data were collected from our digital records and are available on request.
